# Transcriptome Analysis Identifies LINC00152 as a Biomarker of Early Relapse and Mortality in Acute Lymphoblastic Leukemia

**DOI:** 10.3390/genes11030302

**Published:** 2020-03-13

**Authors:** Diego Alberto Bárcenas-López, Juan Carlos Núñez-Enríquez, Alfredo Hidalgo-Miranda, Fredy Omar Beltrán-Anaya, Didier Ismael May-Hau, Elva Jiménez-Hernández, Vilma Carolina Bekker-Méndez, Janet Flores-Lujano, Aurora Medina-Sansón, Edna Liliana Tamez-Gómez, Víctor Hugo López-García, José Ramón Lara-Ramos, Nora Nancy Núñez-Villegas, José Gabriel Peñaloza-González, Luz Victoria Flores-Villegas, Raquel Amador-Sánchez, Rosa Martha Espinosa-Elizondo, Jorge Alfonso Martín-Trejo, Martha Margarita Velázquez-Aviña, Laura Elizabeth Merino-Pasaye, María Luisa Pérez-Saldívar, David Aldebarán Duarte-Rodríguez, José Refugio Torres-Nava, Beatriz Cortés-Herrera, Karina Anastacia Solís-Labastida, Ana Itamar González-Ávila, Jessica Denisse Santillán-Juárez, Alejandra Jimena García-Velázquez, Haydee Rosas-Vargas, Minerva Mata-Rocha, Omar Alejandro Sepúlveda-Robles, Juan Manuel Mejía-Aranguré, Silvia Jiménez-Morales

**Affiliations:** 1 Programa de Doctorado, Posgrado en Ciencias Biológicas, Universidad Nacional Autónoma de México, Mexico City 04510, Mexico; d.a.barcenas@outlook.com; 2 Unidad de Investigación Médica en Epidemiologia Clínica, UMAE Hospital de Pediatría “Dr. Silvestre Frenk Freund”, Centro Médico Nacional “Siglo XXI”, Instituto Mexicano del Seguro Social, Mexico City 06720, Mexico; jcarlos_nu@hotmail.com (J.C.N.-E.); janetflores22@yahoo.com.mx (J.F.-L.); maria_luisa_2000_mx@yahoo.com (M.L.P.-S.); turunci@gmail.com (D.A.D.-R.); 3 Laboratorio de Genómica del Cáncer, Instituto Nacional de Medicina Genómica (INMEGEN), Mexico City 14610, Mexico; ahidalgo@inmegen.gob.mx; 4 Programa de Doctorado en Ciencias Biomédicas, Universidad Nacional Autónoma de México, Mexico City 04510, Mexico; frebeltran@hotmail.com; 5 Programa de Maestría en Investigación Clínica Experimental en Salud, Universidad Nacional Autónoma de México, Mexico City 04510, Mexico; didier_may@outlook.com; 6 Servicio de Hematología Pediátrica, Hospital General “Gaudencio González Garza”, Centro Médico Nacional “La Raza”, IMSS, Mexico City 02990, Mexico; elvajimenez@yahoo.com (E.J.-H.); nanuvi_2401@yahoo.com.mx (N.N.N.-V.); 7 Unidad de Investigación Médica en Inmunología e Infectología, Hospital de Infectología “Dr. Daniel Méndez Hernández”, Centro Médico Nacional “La Raza”, IMSS, Mexico City 02990, Mexico; bekkermendez@yahoo.com; 8 Servicio de Hemato-Oncologia, Hospital Infantil de México Federico Gómez, Secretaria de Salud (SS), Mexico City 06720, Mexico; auroramedina@aol.com.mx; 9 Servicio de Hemato-Oncología Hospital Infantil de Tamaulipas, Secretaría de Salud (SS), Cd. Victoria Tamaulipas 87070, Mexico; lilianatamez@hotmail.com; 10 Servicio de Ortopedia Pediátrica, Hospital Infantil de Tamaulipas, Secretaría de Salud (SS), Cd. Victoria Tamaulipas 87070, Mexico; ortopediatra81@gmail.com; 11 Departamento de Genética, Hospital Infantil de Tamaulipas, Secretaría de Salud (SS), Cd. Victoria Tamaulipas 87070, Mexico; lara_mayor@hotmail.com; 12 Servicio de Onco-Pediatría, Hospital Juárez de México, Secretaría de Salud (SS), Mexico City 07760, Mexico; penaloza_6@yahoo.es (J.G.P.-G.); m_mvelazquez@yahoo.com.mx (M.M.V.-A.); 13 Servicio de Hematología Pediátrica, Centro Médico Nacional “20 de Noviembre”, Instituto de Seguridad y Servicios Sociales de los Trabajadores del Estado (ISSSTE), Mexico City 03100, Mexico; victoriabanco@yahoo.com.mx (L.V.F.-V.); sketch0712@gmail.com (L.E.M.-P.); 14 Hospital General Regional 1 “Dr. Carlos McGregor Sánchez Navarro”, IMSS, Mexico City 03103, Mexico; dibs_amador@hotmail.com (R.A.-S.); itamarga@hotmail.com (A.I.G.-Á.); 15 Servicio de Hematología Pediátrica, Hospital General de México “Dr. Eduardo Liceaga”, Secretaría de Salud (SS), Mexico City 06720, Mexico; rmespinosa1605@hotmail.com (R.M.E.-E.); beatrizcortes101087@gmail.com (B.C.-H.); 16 Servicio de Hematología Pediátrica UMAE Hospital de Pediatría “Dr. Silvestre Frenk Freund”, Centro Médico Nacional “Siglo XXI”, IMSS, Mexico City 06720, Mexico; jorge.martintr@imss.gob.mx (J.A.M.-T.); kas_anastacia@yahoo.com (K.A.S.-L.); 17 Servicio de Oncología, Hospital Pediátrico de Moctezuma, Secretaria de Salud del D.F., Mexico City 15530, Mexico; torresoncoped@live.com.mx; 18 Servicio de Hemato-Oncología Pediátrica, Hospital Regional No. 1 de Octubre, ISSSTE, Mexico City 07300, Mexico; jessydenise22@hotmail.com (J.D.S.-J.); ale.garciavelazquez@gmail.com (A.J.G.-V.); 19 Unidad de Investigación en Genética Humana, UMAE Hospital de Pediatría “Dr. Silvestre Frenk Freund”, Centro Médico Nacional “Siglo XXI”, IMSS, Mexico City 06720, Mexico; hayrov@gmail.com (H.R.-V.); mine_mr@hotmail.com (M.M.-R.); sero__82@hotmail.com (O.A.S.-R.); 20 Coordinación de Investigación en Salud, IMSS, Mexico City 06720, Mexico

**Keywords:** acute lymphoblastic leukemia, long noncoding RNA, *LINC00152*, *LINC001013*, early relapse, microarray expression analysis

## Abstract

Evidence showing the role of long non-coding RNAs (lncRNAs) in leukemogenesis have emerged in the last decade. It has been proposed that these genes can be used as diagnosis and/or prognosis biomarkers in childhood acute lymphoblastic leukemia (ALL). To know if lncRNAs are associated with early relapse and early mortality, a microarray-based gene expression analysis in children with B-lineage ALL (B-ALL) was conducted. Cox regression analyses were performed. Hazard ratios (HR) and 95% confidence intervals (95% CI) were calculated. *LINC00152* and *LINC01013* were among the most differentially expressed genes in patients with early relapse and early mortality. For *LINC00152* high expression, the risks of relapse and death were HR: 4.16 (95% CI: 1.46–11.86) and HR: 1.99 (95% CI: 0.66–6.02), respectively; for *LINC01013* low expression, the risks of relapse and death were HR: 3.03 (95% CI: 1.14–8.05) and HR: 6.87 (95% CI: 1.50–31.48), respectively. These results were adjusted by NCI risk criteria and chemotherapy regimen. The lncRNA–mRNA co-expression analysis showed that *LINC00152* potentially regulates genes involved in cell substrate adhesion and peptidyl–tyrosine autophosphorylation biological processes. The results of the present study point out that *LINC00152* could be a potential biomarker of relapse in children with B-ALL.

## 1. Introduction

Acute leukemia is the most common pediatric malignancy worldwide, representing from one-third of all childhood cancers registered in economically developed countries to nearly 50% in developing ones [1,2,3]. B-lineage acute lymphoblastic leukemia (B-ALL) is the most frequent leukemia subtype, which displays one of the highest incidences and mortality rates in the Mexican population in comparison with other ethnic groups [2,4]. On the other hand, relapses occurring during early stages of treatment have been documented as one of the main obstacles to achieve better survival rates in Mexican children despite the use of the same chemotherapy protocols of developed countries, where survival rates are higher than 90% at 5-years of follow-up [4,5]. Therefore, better clinical and biological prognosis stratification before treatment initiation is mandatory.

Long non-coding RNAs (lncRNAs) transcripts are emerging as potential diagnosis and/or prognosis biomarkers in diverse human diseases, including hematological malignancies [6,7,8,9,10]. They are defined as transcripts longer than 200 nucleotides, and these genes represent the largest set of non-coding RNAs in the human transcriptome. They also regulate gene expression at the transcriptional, post-transcriptional, and epigenetic levels through their interaction with DNA, RNA, and proteins [9]. In this regard, different studies conducted in children with ALL and some performed in animal models reported that the lncRNAs signatures might be useful to identify molecular ALL subtypes and novel prognosis biomarkers of the disease [11,12,13,14,15,16]. As an example, from a whole-transcriptome deep sequencing analysis of the Jurkat leukemic T-cell line, an overexpression of T-ALL-R-LncR1 associated with this ALL subtype was found [11]. As well, Fernando et al. studying 44 ALL children by microarray observed that *BALR-2* overexpression increases proliferation, reduces apoptosis, and correlates with poor overall survival (OS) and a low response to prednisone treatment [7]. Another study of whole transcriptome sequencing involving 56 ALL cases reported that the expression inhibition of the lncRNAs *RP11–624C23.1*, *RP11–203E8*, and *RP11–446E9* could confer advantages to ALL cells by influencing biological processes such as apoptosis, cell proliferation and migration, and DNA damage response and resistance to genotoxic stress [14]. To our knowledge, a limited number of studies have explored the association between the expression of lncRNAs and the risk of relapse in children with B-ALL [16,17]. One of the most recent works was performed in 25 pediatric preB-ALL patients, which, by RNA sequencing and DNA methylation, found a set of *DUX4*, Ph-like specific near haploid/high hyperdiploid subtypes and relapse-specific lncRNAs [16]. The aim of the present study is to investigate whether lncRNA expression profiles associate with the development of early relapses and early deaths in Mexican children with B-ALL.

## 2. Materials and Methods

### 2.1. Collection of Human Samples

As part of the Mexican Interinstitutional Group for the Identification of the Causes of Childhood Leukemia (MIGICCL), we carried out a multicenter cohort study which included patients under 18 years old diagnosed with preB-ALL from years 2014 to 2016 and followed-up for at least 36 months after diagnosis. Eight public hospitals located in Mexico City participated.

Children with Down syndrome, other ALL (e.g., T-cell, mixed lineage), and patients living outside Mexico City during treatment were not eligible. Diagnosis confirmation was performed by pediatric hematologists/oncologists based on the morphology and immunophenotype of leukemic cells. Bone marrow (BM) samples were obtained before treatment initiation, and all patients received their chemotherapy treatment in the same hospital where they were diagnosed with ALL.

All clinical data (sex, age at diagnosis, white blood cell count, immunophenotype, risk classification group, and chemotherapy regimen) were registered from the patients’ medical records. The National Cancer Institute (NCI) risk stratification was employed (standard risk = from 1 to 9.99 years of age or white blood cell (WBC) count < 50 × 10^9^/L, and high risk ≤ 1 or ≥10 years of age and/or WBC ≥ 50 × 10^9^/L).

An early relapse was considered when ≥5% leukemic blasts were noted in a bone marrow sample during the first 36 months after having achieved complete remission (CR). The existence of lymphoblasts on smears of cerebrospinal fluid (mononuclear cell count ≥ 5/mL) accompanied (or not) with cranial nerve paralysis-determined central nervous system (CNS) relapse following the first CR. Early mortality was defined as the patient’s death during the first 24-months.

The National Scientific Research and Ethics Committee of the Mexican Institute of Social Security approved the protocol with the number R-2013-785-068. Written informed consent was obtained from the children’s parents and patients ≥8 years old gave their assent (when possible) to be enrolled in the present study.

### 2.2. Total RNA Isolation, Microarray Processing, and Data Normalization

Patients whose BM were >70% in blast cells at diagnosis were included. After BM aspiration, samples were processed, and leukemic blasts were treated with TRIzol reagent (Invitrogen Life Technologies, Carlsbad, CA, USA). Total RNA isolation and expression analysis were performed as previously described [18]. Briefly, RNA was purified from BM-derived leukemic blasts, then quantified using a Nanodrop spectrophotometer ND1000 (Thermo Fisher Scientific, Waltham, MA, USA), and RNA quality was verified using Agilent Bioanalyzer 2100 (Agilent Technologies, Santa Clara, CA, USA). Samples with an RNA integrity number (RIN) ≥ 7.0 were considered for microarray analyses. Affymetrix GeneChip Human Transcriptome Array 2.0 (HTA 2.0, Affymetrix Inc., Santa Clara, CA, USA) and 200 ng of total RNA were used according to the manufacturer’s recommendations (Affymetrix Inc., Santa Clara, CA, USA). The HTA 2.0 array was designed to interrogate coding and non-coding transcripts, including lncRNAs. The RNA was converted into cDNA and labeled with the WT Plus Reagents Kit (Affymetrix, Santa Clara, CA, USA) mixed with the Poly-A RNA spike-in controls and hybridized on the array according to the manufacturer’s instructions (each hybridization was performed in duplicate). Arrays were washed, stained and scanned using a GeneChip Scanner 3000 7G (Affymetrix, Santa Clara, CA, USA), followed by the transformation of fluorescence data into CEL files using the Affymetrix GeneChip Command Console (AGCC) software. Background correction, probe sets signal integration, and quantile normalization were performed through the robust multi-array average (RMA) algorithm, also implemented in Affymetrix Expression Console (ECS) software, and following the protocols established by the manufacturer (Affymetrix Inc., Santa Clara, CA, USA).

### 2.3. mRNA and lncRNA Transcriptional Profiling Analysis

To identify mRNA and lncRNA profiles and potential lncRNA associated with early relapse and mortality, a supervised analysis was conducted, including both coding and non-coding probes (44,699 and 22,829 transcript clusters, respectively). To identify lncRNAs annotated with Ensembl, words like processed_transcript, lincRNA, antisense, non_coding, sense_intronic, ncRNA_host, sense_overlapping and 3prime_overlapping_ncrna, uncharacterized LOC, LINC-, FLJ, (Antisense: AS and -Host gene: HG) were used. Non-annotated probes from the array were manually annotated by searching them on the UCSC genome browser (https://genome.ucsc.edu/) and LNCipedia (https://lncipedia.org/) webpages. Probes mapping pseudogenes were discarded.

BM samples at the time of diagnosis were classified for analyses if the patient had presented (1) relapse, (2) death during follow-up, and (3) hyperleukocytosis at the time of diagnosis (≥100,000 × mm^3^). To select differentially expressed genes, a threshold fold change (FC) value greater than 1.2 and a false discovery rate (FDR) < 0.05 were used.

### 2.4. Quantitative Real-Time PCR Validation

Quantitative RT–PCR (q–PCR) was performed to validate the expression of the two deregulated lncRNAs identified in the subgroup of children who developed relapse. *LINC00152* (ENSG00000222041) and *LINC01013* (ENSG00000228495) were validated using predesigned TaqMan Gene Expression human assays (Hs03654336_m1 and Hs00395149_m1, respectively) and Universal Master Mix II (Thermo Fisher Scientific, Waltham, MA, USA). Quality control and RNA concentrations were determined by using the NanoDrop 100 spectrophotometer (Thermo Fisher Scientific). cDNA was synthesized from 250 ng of total RNA for each sample using OdT primers and the High-Capacity cDNA Reverse Transcription Kit. Reactions were performed in a final volume of 10 μL under the following conditions: at 95 °C for 10 min, followed by 45 cycles at 95 °C for 15 s and 60 °C for 1 min in a QuantStudio 3 (All Thermo Fisher Scientific, Waltham, MA, USA). Fold changes in expression were calculated by using the 2^−ΔΔCt^ method. Data were normalized using *SCARNA5* as a control reference gene.

### 2.5. lncRNA–mRNA Co-Expression Analysis and Biological Pathways Analysis

To gain insight into the biological relevance of the lncRNAs associated with relapse, a guilt-by-association approach was followed to investigate their relationship with different biological pathways [19]. Normalized gene expression of mRNAs and lncRNAs of the 62 arrays (23 ER-ALL and 39 NR-ALL cases) were used to construct a correlation matrix (Spearman correlation and *p*-value), including only the differentially expressed lncRNAs and mRNAs (FC = 1.5, *p* < 0.05 and FDR ≤ 0.05) genes. For each of the differentially expressed lncRNAs, mRNAs according to their correlation coefficient and *p*-value were ranked. A significant correlation between lncRNAs and mRNAs was considered when Spearman correlation was greater than 60% (positive or negative) and the *p*-value was ≤0.0001. The significantly correlated mRNAs were also evaluated by the Gene Ontology (GO) enrichment pathway analysis to define their impact in signaling cell processes.

### 2.6. Validation Cohort

The validation cohort comprised a subset of cases with ALL and Pre-B immunophenotype, aged 1 to 18 years, included in the MIGICCL study between 2010 and 2013, and who had an available, good quality sample for genetic analysis. Patients who relapsed early (cases) were retrospectively identified in the database of the MIGICCL, and they were frequency-matched with patients without relapse (controls) according to age at diagnosis (±18 months), chemotherapy protocol, NCI risk stratification, the same time of follow-up, and presence/absence of gene fusions. Two controls per case were selected when possible.

### 2.7. Statistical Analysis

To compare demographic, clinical and molecular characteristics between groups, the chi-square test or Fisher exact tests were calculated when appropriate. In the discovery cohort, to classify the low and high gene expression, the cutoff value was determined according to the median. This because gene expression values were not normally distributed according to the Shapiro–Wilk test (*p* < 0.05). Nonetheless, gene expression values of lncRNAs in the validation cohort (measured by qRT–PCR) displayed a normal distribution. Thus, to classify the low and high gene expression, the cutoff value was determined according to the mean. Student’s *t*-test was used to compare the means of expression values between groups. Furthermore, survival analyses were carried out using the Kaplan–Meier method. Disease-free survival (DFS) and overall survival (OS) were calculated, and stratification by NCI risk classification was performed. Log-rank tests were obtained; a *p*-value less than 0.05 was considered as statistically significant. Afterwards, Cox regression analyses were performed. Hazard ratios (crude and adjusted) and 95% confidence intervals were obtained in both the discovery and validation cohorts. Adjusting variables were selected considering their well-known clinical prognostic relevance and their association with relapse in the univariate analysis. These variables were age (*p* < 0.001), NCI risk (*p* = 0.004) and chemotherapy regimen (*p* = 0.04, for the St. Jude Total XIIIB) in the discovery cohort. However, considering that the NCI risk classification includes the age of the patients and that we identified a high correlation (48%) between NCI risk classification and age in the discovery cohort, we have only included the NCI risk classification in the final model to avoid a multicollinearity phenomenon. Consequently, the variables considered for adjusting HRs in the discovery cohort were NCI risk classification and the chemotherapy regimen. In the validation cohort, the variable selected for adjusting in the multivariate analysis was gender (*p* = 0.03).

## 3. Results

### 3.1. Clinical and Demographic Features of the Studied Population

#### 3.1.1. Discovery Cohort

A total of sixty-two BM samples collected at diagnosis from children with de novo B-ALL. Thirty-four (54.8%) patients were male; the median age of the population was 97 months (range: 14–195 months). Seven (11.3%) patients were *BCR-ABL1*, 12 (19.35%) were *ETV6-RUNX1*, 4 (6.45%) were *TCF3-PBX1* positive, and 39 were negative to these four common fusion genes. Twenty-three (37.1%) patients with relapse and 39 (62.9%) with no-relapse were analyzed. The median of the follow-up of patients was 36 months (range: 3.6–50.1 months) after diagnosis confirmation. Relapses occurred in a range from 2 to 36 (mean = 13) months after achieving complete remission.

Of relapsed cases, isolated BM relapse occurred in 17 (73.9%), three (13.04%) had isolated central nervous system (CNS) relapse, and three (13.04%) presented combined relapse (two: BM + CNS and one: BM + testicular). Fifteen (65%) patients relapsed and died early. The mean survival time in the group of patients without relapse was 36 (30 to 43 months); meanwhile, in patients who suffered early relapse, it was 23 (4 to 50) months. There were no statistically significant differences between groups with or without relapse regarding variables such as gender, leukocyte count in peripheral blood at diagnosis, BM, blast percentage at diagnosis, and gene rearrangement frequencies. Significant differences were noted for age at diagnosis, NCI risk categories, and death (*p* = 0.0001, 0.004, and 0.0001, respectively; Table 1).

#### 3.1.2. Validation Cohort

In the validation cohort, a total of 69 B-ALL cases diagnosed between 2010 to 2013 were included. The median follow-up time was 34.5 months (range: 9.9–84.7 months). Of these, 20 (25.75%) and 49 (74.25%) patients corresponded to patients with early relapse and patients without relapse, respectively. In contrast with the discovery cohort, statistically significant differences were observed regarding gender and peripheral WBC count at diagnosis. Clinical and demographic characteristics of the validation cohort are displayed in Table S1.

### 3.2. LINC00152 and LINC01013 Were Abnormally Regulated in Patients with Early Relapse and Early Death

To identify differentially expressed lncRNAs in patients with poor outcomes, we considered relapse and death as independent events. We detected 57 lncRNAs differentially expressed (*p* < 0.05) between early relapse vs. no relapse groups, of which 27 were up-regulated (FC = 1.22 to 8.47) and the remainder 30 down-regulated (FC = −1.24 to −5.88). *LINC00152* and *LINC01013* (FC = 2.24, *p* = 0.0005 and FC = −5.88, *p* = 0.0004, respectively) were among the most up- and down-regulated; even so, none of the lncRNA maintained the statistical significance after multiple comparison tests (Table S2A).

Regarding lncRNAs associated with early death, a total of 124 lncRNA were abnormally expressed in cases who died, of which 28 were up-regulated and 96 down-regulated (FC 1.2, *p* < 0.05). To note, the *LINC00152* (FC = 2.03, *p*= 0.0048) and *LINC001013* (FC = −7.79, *p* = 0.0004) were among the most up-regulated and down-regulated genes, respectively (Table S2B).

### 3.3. Up-Regulated lnRNAs in Patients with hyperleukocytosis

Hyperleukocytosis (≥100 × 10^9^/L WBC) is considered one of the most important independent risk predictors of relapse in children with ALL. The analysis conducted to identify lncRNAs associated with hyperleukocytosis exhibited a total of 434 lncRNAs differentially expressed among patients with hyperleukocytosis and patients with a WBC less than 100 × 10^9^/L. Overall, 130 were up-regulated and 304 down-regulated. Some of the lncRNAs that showed the highest expression were *TCONS_00004231* (FC = 13.49, *p* = 0.001), *SOCS2-AS1* (FC = 5.14, *p* = 0.01), and *LOC101927497* (FC = 3.39, *p* = 0.0049) (Table S2C).

### 3.4. Co-Expression of lncRNA-Coding Genes and Biological Pathways Analysis

**Figure 1:**
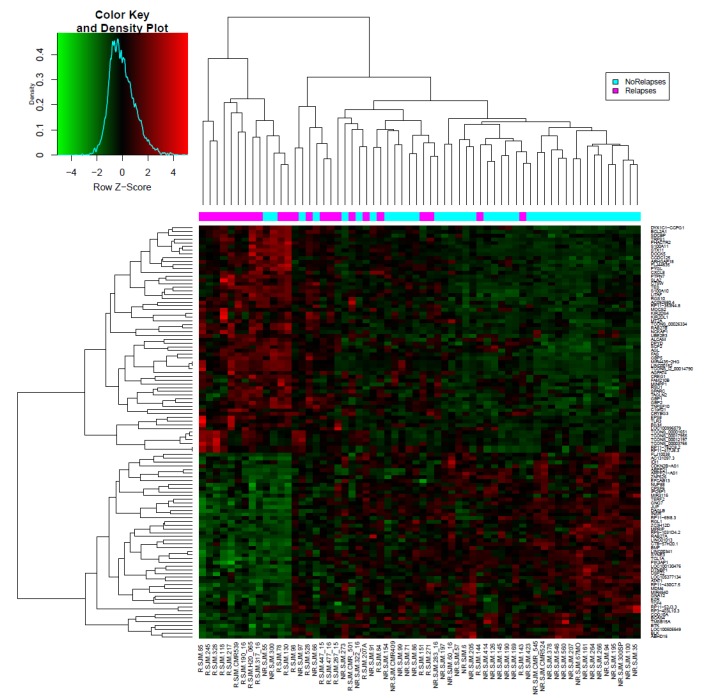
Differentially expressed genes in pediatric patients with acute lymphoblastic leukemia with early relapse vs. non-relapsed cases. Rows display the genes identified, while the columns represent samples (early relapse: pink color and no-relapse: blue). Genes significantly down-regulated are displayed in green and up-regulated genes in red.

To select a set of lncRNAs and coding RNAs that define the profile of patients with early relapse, a supervised-clustering analysis of gene expression using R software (Bioconductor package) was carried out. A total of 111 (27 lncRNAs and 84 coding genes) differential expressed genes (FC = 1.5, *p* < 0.01) among groups with and without relapse were identified (Figure 1).

**Figure 2:**
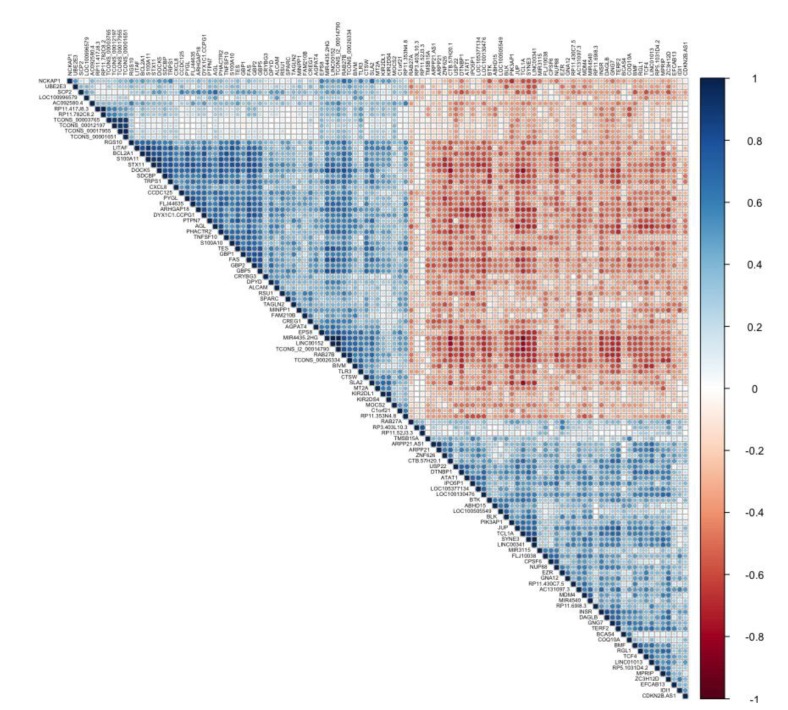
LncRNA–mRNA co-expression. Showing a positive (blue) or negative (brown) correlation between differentially expressed lncRNA and mRNA.

In order to detect potential targets of the lncRNAs differentially expressed in groups, we performed a correlation analysis that included all genes. Pairs showing a positive correlation in their expression patterns are displayed in Figure 2.

The *TCONS_l2_00014790*, *MIR4435.2HG*, *LINC00152*, *TCONS_00026334*, *DYX1C1.CCPG1*, and *LINC01013* were the lncRNAs that showed the highest correlation (Rho 0.6), with most of the abnormally regulated coding genes (*n* = 55, 54, 52, 51, 46, and, 32 genes, respectively) (Table S3). Based on the hypothesis that ncRNA and coding genes positively co-expressed are involved in the same biological pathway, we carried out the GO analysis to explore the potential regulatory mechanism of these genes. Our analysis showed that the co-expression correlation network of altered lncRNA–mRNA in relapse was enriched mainly in substrate cellular adhesion, peptidyl-tyrosine autophosphorylation, peptidyl-tyrosine phosphorylation, and regulation of cell proliferation biological processes. *LINC01013* did not show statistical significance with any biological pathway (Figure S1).

### 3.5. Clinical Association and Survival Analysis

#### 3.5.1. Discovery Cohort

**Figure 3:**
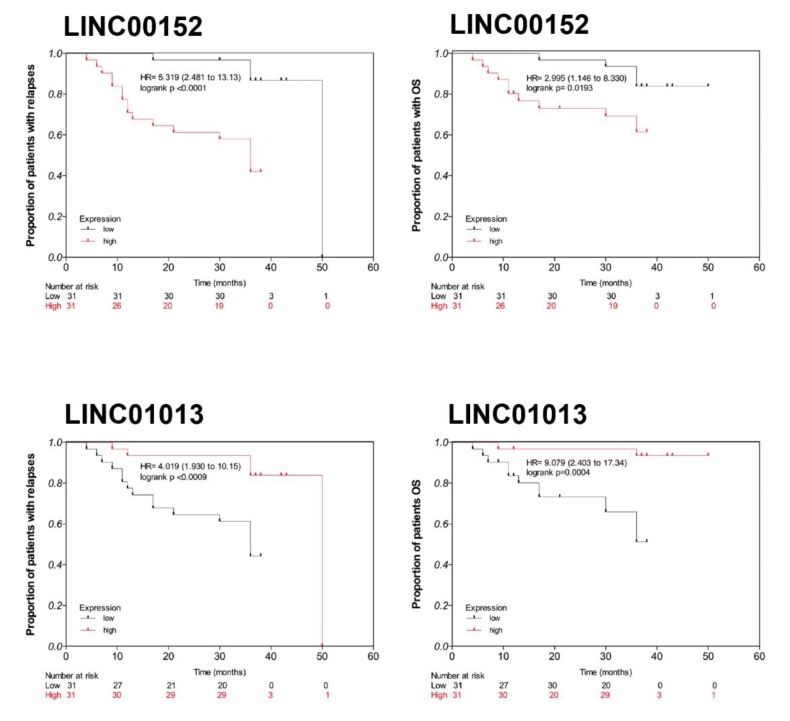
Kaplan–Meier plots (unadjusted) of overall survival for patients with pre-B acute lymphoblastic leukemia. Normalized array expression was used to determine either high (above median) or low (below median) expression of both lncRNAs (*p*-value was calculated with two-sided log-rank test).

Since *LINC00152* and *LINC01013* have been reported as relevant in the hematopoiesis processes and that both genes exhibited co-expression with coding genes that have been reported as cancer-related, the expression of both genes was used to evaluate their clinical significance. The association among the *LINC00152* and *LINC01013* expression (high or low, according to the median of their expression level) with early relapse and death revealed significant differences in hazard ratios (HRs). In the case of *LINC00152*, its high expression was associated with a higher risk of early relapse (HR = 5.319, 95% CI = 2.48–13.13, log-rank test *p* < 0.0001) and early death (HR = 2.995, 95% CI = 1.146–8.33, log-rank test *p* = 0.0193). Meanwhile, *LINC01013* low expression was also associated with early relapse (HR = 4.019, 95% CI = 1.93–10.15 log-rank test *p* < 0.0009) and death (HR = 9.079, CI = 2.40–17.34 log-rank test *p* = 0.0004) (Figure 3).

Furthermore, when HRs were adjusted by NCI risk classification and chemotherapy regimen, the associations were the following: (a) for *LINC00152* high expression, the risks of relapse and death were 4.16 (95% CI: 1.46–11.86) and 1.99 (95% CI: 0.66–6.02), respectively, and (b) for *LINC01013* low expression, the risks of relapse and death were HR: 3.03 (95% CI: 1.14–8.05) and HR: 6.87 (95% CI: 1.50–31.48), respectively.

Survival analysis stratified by NCI risk categories showed that in the standard-risk group presenting a low expression level of *LINC00152*, the DFS rate was 93%, while in those having a high expression of *LINC00152*, the disease-free survival (DFS) rate was 62.5%. For those patients classified as high risk according to NCI and having a low expression of *LINC00152*, the DFS was 75%, whereas, in the subgroup presenting high levels of *LINC00152* expression, the DFS was 34.8%. These results were statistically significant (log-rank *p* = 0.003).

When these associations were explored for the *LINC1013*, standard-risk patients presenting high expression levels of this gene showed a DFS rate of 85.7%, whereas in those cases having a low expression of *LINC01013*, the DFS was 77.8% (*p* = 0.33). In the group of patients classified as high risk and having a high expression of *LINC01013*, the DFS was higher (76.5%) in comparison to children with low expression levels of this gene (31.8%; *p* = 0.01).

It is worth noting that OS rate in patients classified as standard-risk but having a low expression level of *LINC00152* was 93.3%, whereas in those children having a high expression of this gene, the OS was 87.5% (*p* = 0.62). Furthermore, for patients classified as high risk (by NCI criteria) presenting a low expression of *LINC00152*, the OS was 75.0%, and the subgroup with high levels of *LINC00152* expression, the OS was 30.4% (*p* = 0.17).

To note, in the standard-risk group with a high expression level of *LINC01013*, none of the patients died during follow-up, whereas in those having a low expression of this gene, the OS was 77.8% (*p* = 0.07). For patients classified as high risk presenting a high expression of *LINC01013*, the OS was higher compared to the subgroup with low expression levels of *LINC01013* (88.2% and 33.3%, respectively; *p* = 0.02).

#### 3.5.2. Validation Cohort

To validate the results identified in the discovery cohort, the expression of *LINC00152* and *LINC01013* was analyzed by qRT–PCR in an independent cohort (validation cohort) comprised of 69 children with ALL (20 with relapse and 49 without relapse). Given the fact that we used qRT–PCR to evaluate the expression levels of both genes and that the values obtained were normally distributed, low/high expression was categorized according to the mean of expression values (mean: 0.00002218290410 SD = 0.000032891429075 and 0.0363975856084 SD = 0.087986926396332 for *LINC00152* and *LINC01013*, respectively). Remarkably, the expression of both genes exhibited the same tendency as the discovery cohort. The expression levels of *LINC00152* were increased, while they were decreased for *LINC01013* in patients who developed early relapse. Notwithstanding, the statistical significance remained only to *LINC00152* (*p* = 0.03; Figure S2). No associations were identified between levels of expression of *LINC01013* and relapse (*p* = 0.16) or death (*p* = 0.43) in the validation cohort.

**Figure 4:**
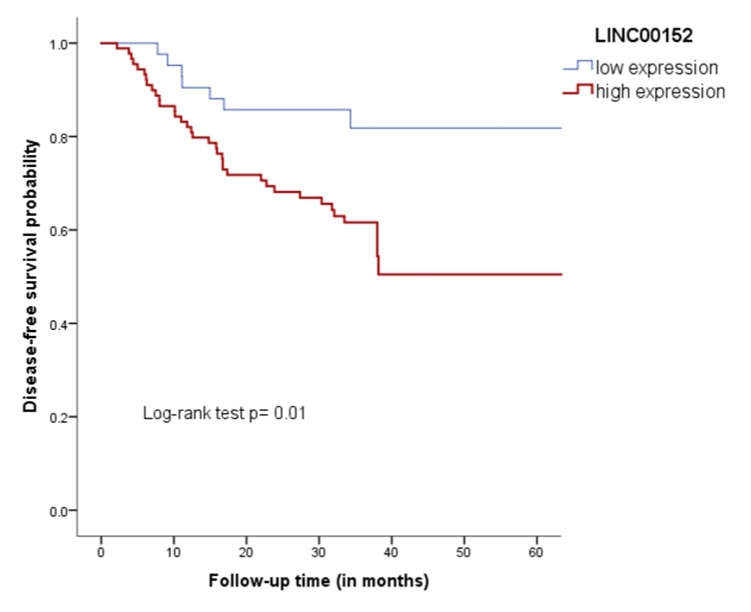
Survival analysis between a low/high expression of LINC00152 with relapse.

In a Kaplan–Meier analysis, a lower DFS rate (50.5%) was observed in the group with a high expression of *LINC00152* in comparison to patients with a low expression (81.8%) (Figure 4). Nonetheless, non-significant differences were observed between levels of expression of *LINC00152* and OS (*p* = 0.06). Similarly, low levels of expression of *LINC01013* were not associated with DFS or OS rates (*p* = 0.22 and *p* = 0.59, respectively).

Furthermore, an increased risk of relapse was observed for the *LINC00152* (cHR: 1.90; 95% CI: 0.75–4.80); however, the confidence intervals were imprecise. When the association was adjusted by gender, the aHR was 1.71 (95% CI: 0.67–4.36). Likewise, no statistically significant association was identified between this gene and death (cHR: 0.98; 95% CI: 0.20–4.87).

By stratifying NCI risk groups in this cohort, none of the patients from the standard-risk group with a low expression level of *LINC00152* relapsed during follow-up, while in those having a high expression of *LINC00152* the DFS rate was 58.4%. Furthermore, for patients classified as high risk according to NCI criteria who presented a low expression of *LINC00152*, the DFS was 74.9%, whereas in the subgroup with high levels of expression for this gene, the DFS was 45.6%. Our results suggest that a high expression of *LINC00152* is associated with lower DFS rates regardless of the NCI risk category (*p* = 0.007). This finding was more evident in the standard-risk group. No associations were observed between a low expression of *LINC1013* and DFS (*p* = 0.30) across NCI groups.

Otherwise, OS analysis showed that in the standard-risk group with a low expression level of *LINC00152*, none of the patients presented early death during follow-up, while in those having a high expression of *LINC00152*, the OS frequency was 93.4%. For patients classified as high risk presenting a low expression of *LINC00152*, the DFS was 89.7%; meanwhile, the DFS was 66.1% in the subgroup with high expression levels for this gene. Thus, our findings reveal that a high expression of *LINC00152* is associated with lower OS rates among NCI categories (*p* = 0.03), being more evident in the high-risk group. No associations were observed between a low expression of *LINC1013* and OS (*p* = 0.28) rates across NCI groups.

## 4. Discussion

So far, it is predicted that more than 90% of the human genome is actively transcribed, of which only 2% of this genome is coded into proteins and the remaining is transcribed into non-coding genes, mainly lncRNAs [16]. Over the last few years, by using high-throughput technologies for measuring the lncRNAs expression in different cell types (either with normal or pathological conditions), multiple studies have been conducted. Although the molecular mechanisms of most lncRNAs are unknown, increasing evidence suggests that these genes are important regulators of the expression of coding-genes [9]. Among the molecular mechanisms described for these genes are modifications to the spatial conformation of chromosomes, chromatin and DNA modifications, RNA transcription, pre-mRNA splicing, mRNA degradation, mRNA translation, or by competing for microRNA binding [9,20]. Thus, changes in lncRNA expression may contribute to the development and progression of human diseases such as ALL, the most common type of childhood cancer around the globe [1,2,6,9,10]. Despite the advances achieved in the treatment of this disease, relapses still occur in approximately 20% of patients, and it is one of the main obstacles to gain better survival rates in developed populations. Relapse is even greater in Mexican children with ALL (17% during the early stages of treatment) [4].

### 4.1. LINC00152 and LINC01013 Expression in Acute Lymphoblastic Leukemia

Comparing BM at diagnosis from early relapse vs. no relapse cases, we identified 57 lncRNA differentially expressed, some of which had been previously reported in human tumors (*LOC100130476*, *LINC00152*, *LINC01013*, *MIR4435–2HG*) [21,22,23,24,25,26,27]. For instance, it has been referred that down-regulation of *LOC100130476* inhibits proliferation and invasiveness of cancer cells in esophageal squamous cell carcinoma and gastric cardia adenocarcinoma [23,24]. *LINC00152* was abnormally regulated in gastric, pancreatic, hepatic colon, and breast cancer tumors [21,22,26,27,28,29]; meanwhile, the *LINC01013* was found to be involved in anaplastic large-cell lymphoma [29]. In our study, the *LINC00152* was one of the most up-regulated lncRNAs in pre-B ALL cases suffering an early relapse. Additionally, the high expression of *LINC00152* correlated with a high risk for relapse and death.

*LINC00152*, also known as *CYTOR* (long non-coding RNA cytoskeleton regulator RNA), was recently identified as a cancer-related lncRNA that plays oncogenic roles in several types of cancer [27,30]. A recent meta-analysis exploring the clinical significance and prognosis role of this gene found that an elevated expression of *LINC00152* is associated with metastasis and poor survival in human cancer [27,30]. Additionally, it has been reported that *LINC00152* confers resistance to oxaliplatin in colon cancer [31]. The role of *LINC00152* in biological and pathological processes are continuously being identified, and its location in the nucleus and cytoplasm suggests their involvement in transcription, signaling, and intracellular trafficking [32,33]. Experimental evidence shows that it also regulates cancer-related genes through diverse mechanisms, including epigenetic modifications, miRNAs, and protein interactions [26,33,34]. Data of the present work show that *LINC00152* co-expressed genes are involved in substrate cellular adhesion and peptidyl-tyrosine autophosphorylation biological processes. In vitro and in vivo studies exposed that *LINC00152* promotes tumor progression and epithelial-to-mesenchymal transition process in colon cancer and glioblastoma tumors, respectively, trough modulating the LINC00152/miR-193a-3p/ERBB4/AKT signaling pathway and inhibiting miR-107 and miR-16 expression [31,34,35]. In addition, in glioblastoma, *LINC00152* overexpression correlated with poor prognosis and an increased invasion through a 3′-hairpin structure [36]. We found that *LINC00152* expression correlates with the *MIR4435–2HG* expression (Rh0 = 1). Both genes were differentially expressed between early relapse and no relapse cases (FC = 2.22, *p* = 0.0007 vs. FC = 2.24, *p* = 0.0005, respectively). To note, a sequencing analysis of *LINC00152* revealed a high similarity with *MIR4435–2HG*, its paralog gene partner [33]. *MIR4435–2HG* deregulation has been associated with poor prognosis in breast cancer, tumor progression in gastric cancer tissues, and with histological grades and lymph node metastasis in lung cancer [31,37,38]. Functional analysis demonstrated that *MIR4435–2HG* action in cancer could be by its interaction with β-catenin, which promotes cell proliferation and invasion [38]; however, no data regarding *MIR4435–2HG* and ALL have been reported.

To the best of our knowledge, the role of *LINC01013* in childhood acute leukemia had not been explored; nevertheless, data about the relevance of this gene in hematological malignancies had been given in anaplastic large cell lymphoma (ALCL). *LINC01013* was over-expressed in ALCL in comparison with adjacent noncancerous, and in vitro studies demonstrated that its up-regulation enhanced tumor cell invasion through the induction of snail-fibronectin activation cascade [29]. *LINC01013* was downregulated in our cases with early relapse, in contrast with ALCL findings. The discrepancies between both studies could be explained by a dual (oncogene and gene suppressor) role of *LINC01013* in cancer. Dual roles have been observed in other lncRNAs; for instance, *HOXA11-AS*, which is up-regulated (e.g., breast cancer, osteosarcoma, glioma, hepatocarcinoma), but down-regulated (glioblastoma, colorectal cancer, and ovarian cancer) in several types of cancer [39].

Our co-expression analysis revealed that *LINC00152* regulates genes involved in substrate cellular adhesion processes, one of the main biological mechanisms associated with relapse and chemoresistance in ALL. Chemoresistance has been addressed in several studies as one of the most important mechanisms of relapse and death in ALL [40,41,42]. Taking together, these data and our findings regarding the association between *LINC00152* high expression and the high risk of relapse suggest that *LINC00152* could be potentially involved in chemoresistance; however, functional studies are required to prove these remarks.

### 4.2. LncRNAs in Hyperleukocytosis

In our study, *SOCS2-AS1* and *LOC101927497* genes were differentially expressed in patients who died and presented hyperleukocitosis in peripheral blood at the time of diagnosis confirmation. Data from prostate cancer suggest that *SOCS2-AS1* lncRNA is involved in the disease by promoting androgen signaling due to the modulation of the epigenetic control for androgen receptors target genes [43]. Regarding *LOC101927497* and its functional role in malignantly transformed human gastric epithelial cells, it was found that this ncRNA can suppress the proliferation and migration of human gastric epithelial T-cells in vitro by interacting with miR-574–5p. Thus, it was suggested that this gene is an lncRNA suppressor gene in human gastric epithelial cells [44]. To date, no studies have been conducted to identify lncRNAs in patients with hyperleukocytosis in any type of leukemia.

### 4.3. LINC00152 and LINC01013 as Potential Biomarkers of Poor Outcomes in Acute Lymphoblastic Leukemia

The microarray expression analysis showed that *LINC00152* and *LINC01013* were upregulated and downregulated in ER-ALL cases, respectively. Using qRT–PCR, we validated these findings in a subset of patients from the discovery cohort (data not shown).

In addition, we sought to validate microarrays results by qRT-PCR in an independent population (validation cohort). Statistically significant differences in the association between the expression levels of *LINC00152* but not to *LINC01013* and relapse were observed. The purpose of having made a frequency-match instead of analyzing available data in the validation cohort was to control the effect of variables that are currently used in risk stratification and treatment assignment of children with B-ALL; for instance, age of patients, NCI risk classification, and gene rearrangements. Furthermore, given the different protocols used in the hospitals, we have decided to match by chemotherapy regimen. As displayed in Table S1, non-significant differences were observed in these matching variables between relapse and no-relapse groups. The only variable that showed significant differences among the studied groups was the gender of patients. Then, this variable was selected for adjusting the HRs in the validation cohort. Even though the sample size of the discovery and the validation cohorts were similar, there is still clinical and molecular heterogeneity between them, as well as the chemotherapeutic protocols used by the participants’ institutions, support therapy, and nutritional status that can influence our results [45]. Therefore, statistical differences between discovery and validation cohorts are probably due to biological reasons.

Other limitations of our work include the small sample size, which, in addition to the high molecular heterogeneity of the ALL, influences the statistical power. Furthermore, we analyzed BM from patients who have >70% (at diagnosis) of leukemia cells, so normal hematopoietic cells could act as a confounding factor. To overcome this problem, blast cells should be sorted. Otherwise, we do not know if the stability of the lncRNAs differs under normal and pathological conditions during the evolution of the disease or their association with treatment response [46]. In this regard, it is necessary to investigate whether these genes have an independent prognostic role regarding the minimal residual disease (MRD) status of ALL patients with relapse; however, MRD is not currently performed in our participating hospitals.

To our knowledge, no previous studies have evaluated the relationship between these genes and the risk of relapse or death in ALL cases. Notwithstanding, *LINC00152* and *LINC01013* expression have been correlated with poor prognosis in diverse human cancer types, including acute myeloid leukemia [27,29,47], supporting the potential role of both genes in ALL as risk predictors of poor outcomes. Thus, in order to gain a better understanding of the role of *LINC00152* and *LINC01013* as biomarkers associated with relapse and death in children with ALL, the sample size should be increased in further investigations.

## 5. Conclusions

Nowadays, epigenetic factors are considered as one of the most effective mechanisms in the pathogenesis of different malignancies. These factors are reversible elements, which open up the possibility to use them as indicators of the disease, prognosis biomarkers, and important therapy targets. In the present study, the results support the previously reported evidence pointing out that lncRNAs’ deregulated expression is linked to ALL and relapse; however, further studies are needed to endorse the value of these genes as independent biomarkers of early relapses and deaths.

## Figures and Tables

**Table 1:** Clinical characteristics of the children with B-lineage acute lymphoblastic leukemia from the discovery group.

Clinical Characteristics	ALL Children *n* = 62		*p* *
	Early Relapse		
	No	Yes	
	*n* = 39	*n* = 23	
**Gender**	***n* (%)**	***n* (%)**	0.35
Female	19 (48.7)	9 (39.1)	
Male	20 (51.3)	14 (60.9)	
**Age group (years)**			**0.0001**
1–9.99	29 (74.4)	8 (34.8)	
≥10	10 (25.6)	15 (65.2)	
**Age at diagnosis (months)**			0.002 **
Median (min–max)	73 (14–191)	140 (18–208)	
**WBC count at diagnosis (×10^9^/L)**			0.75
<10	13 (33.3)	5 (21.7)	
10–49.99	12 (30.8)	9 (39.1)	
50–99.99	5 (12.8)	4 (17.4)	
≥100	9 (23.1)	5 (21.7)	
**BM blast (%) at diagnosis**			0.75
<90	7 (17.9)	5 (21.7)	
≥90	32 (82.1)	18 (78.3)	
**Gene rearrangement**			0.26
*ETV6-RUNX1*	7 (17.9)	5 (21.7)	
*TCF3-PBX1*	4 (10.3)	0 (0)	
*BCR-ABL1*	5 (12.8)	2 (10)	
Non-detected	23 (59.0)	16 (68.3)	
**NCI risk classification**			**0.004**
Standard	20 (51.3)	4 (14.3)	
High	19 (48.7)	24 (85.7)	
**Relapse site**			-
Isolated BM	-	17 (74)	
Isolated CNS	-	3 (13)	
BM and CNS	-	2 (8.7)	
BM and testicular	-	1 (4.3)	
**Chemotherapy regimen**			
DFCI (reference)	10 (25.6)	2 (8.7)	-
BFM-95	4 (10.3)	2 (8.7)	0.43
St Jude Total XV	16 (41.0)	8 (34.8)	0.3
St Jude Total XIIIB	9 (23.1)	11 (47.8)	**0.04**
**Death**			<**0.0001**
Yes	0 (0)	15 (65.2)	
No	39 (100)	8 (34.8)	

WCB: whole blood count; BM: bone marrow; NCI: National Cancer Institute, NIH, USA; CNS: central nervous system; DFCI:_Dana Farber Cancer Institute 00–01; BFM-95: Berlin–Frankfurt–Munster-95; * chi square or Fisher exact test when appropriate. ** Mann–Whitney U-test.

## Supplementary Materials

The following are available online at https://www.mdpi.com/2073-4425/11/3/302/s1. Figure S1: Biological pathways by guilt-by-association analysis using lncRNA-mRNA co-expression. Figure S2: Quantitative PCR (qPCR) analysis. Relative expression levels of *LINC00152* and *LINC01013* normalized to *SCARNA5*. Table S1: Demographic and clinical characteristics of the validation cohort. Table S2: Differentially expressed lncRNAs among patients with (A) relapse and and (B) death and hyperleukocytosis (C). Table S3: Coding genes displaying the higher correlation values between lncRNAs and mRNAs.
