# Supplementary material for: Transcriptome Analysis Identifies LINC00152 as a Biomarker of Early Relapse and Mortality in Acute Lymphoblastic Leukemia
Source: Genes (Basel). 2020 Mar 13;11(3):302. doi: 10.3390/genes11030302 (PMC7140896; doi:10.3390/genes11030302)
Supplement: Supplementary file 1 [file genes-11-00302-s001.zip › Supp_table_1 .docx]

**Supplementary Table 1:** Demographic and clinical characteristics of children with pre-B acute lymphoblastic leukemia of the validation cohort

| **Clinical Characteristics** | **ALL children**  **n = 69** | | ***P**** |
| --- | --- | --- | --- |
|  | **Early relapse** | |  |
|  | **No**  **n = 49** | **Yes**  **n = 20** |  |
| **Gender**  Female  Male | **n (%)**  31 (63.3)  18 (36.7) | **n (%)**  7 (35)  13 (65) | 0.03 |
| **Age group (years)**  1-9.99  *>*10 | 36 (73.5)  13 (26.5) | 15 (75.0)  5 (25) | 0.089 |
| **Age at diagnosis (months)**  Median (min-max) | 61 (11-174) | 72 (20-197) |  |
| **WBC count at diagnosis**  **(x10^9^/L)**  < 10  10-49.99  50-99.99  ≥ 100 | 22 (44.9)  19 (38.8)  5 (10.2)  3 (6.1) | 5 (25.0)  8 (40.0)  2 (10.0)  5 (25.0) | 0.12 |
| **BM blast (%) at diagnosis**  <90  >90 | 4 (8.2)  45 (91.8) | 2 (10)  18 (90) | 0.80 |
| **Gene rearrangement**  *ETV6-RUNX1*  *TCF3-PBX1*  *BCR-ABL1*  *MLL-AF4*  Non-detected | 6 (12.3)  4 (8.2)  1 (2)  1 (2)  37 (75.5) | 0 (0)  2 (10)  0 (0)  18 (90) | 0.84 |
| **NCI risk classification**  Standard  High | 15 (30.6)  34 (69.4) | 4 (20)  16 (80) | 0.37 |
| **Relapse site**  Isolated BM  Isolated CNS  BM & CNS  BM & testicular  Testis | __  __  __  __  __ | 14 (70)  1 (5)  2 (10)  1 (5)  2 (10) | __ |
| **Death**  Yes  No | 1 (2)  48 (98) | 5 (25)  15 (75) | 0.002 |
| WCB: Whole blood count; BM: bone marrow; NCI: National Cancer Institute, NIH, USA; CNS: central nervous system; VER: very early relapse (relapse occurred a <18 months at diagnosis); ER: early relapse; *Chi square or Fisher exact test when appropriate. | | | |
