# Supplementary material for: Transcriptome Analysis Identifies LINC00152 as a Biomarker of Early Relapse and Mortality in Acute Lymphoblastic Leukemia
Source: Genes (Basel). 2020 Mar 13;11(3):302. doi: 10.3390/genes11030302 (PMC7140896; doi:10.3390/genes11030302)
Supplement: Supplementary file 1 [file genes-11-00302-s001.zip › Supp_figures_1&2.docx]

*
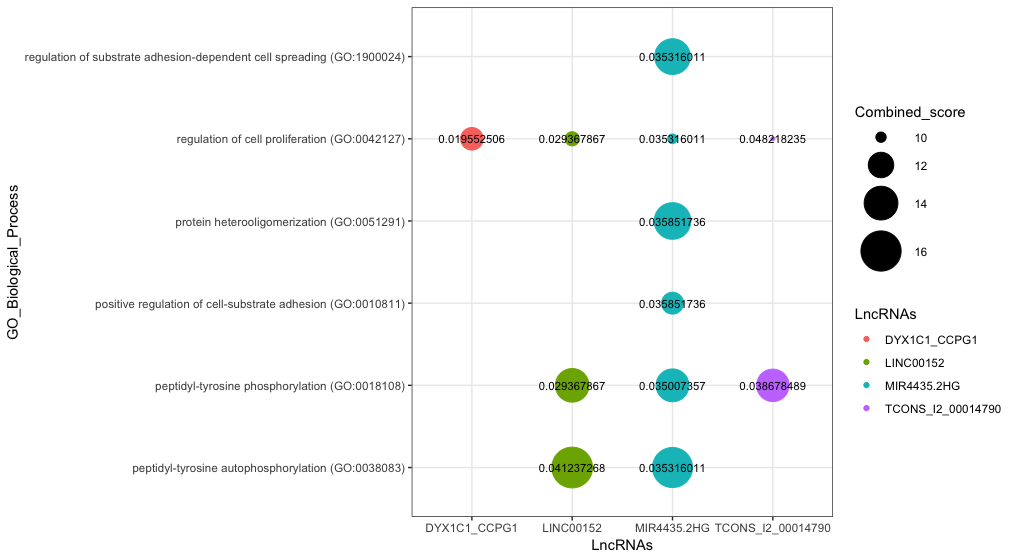
*

Supplementary Figure S1. Biological pathways by guilt‐by‐association analysis using lncRNA‐mRNA co‐expression. Pathway enrichment analysis resulted in significant association with cancer‐related signaling. The y‐axis represents pathways and the x‐axis represents lncRNAs. Bubble chart shows biological pathways enrichment by and the size and color of the bubble represent each gene and the score of each pathway.


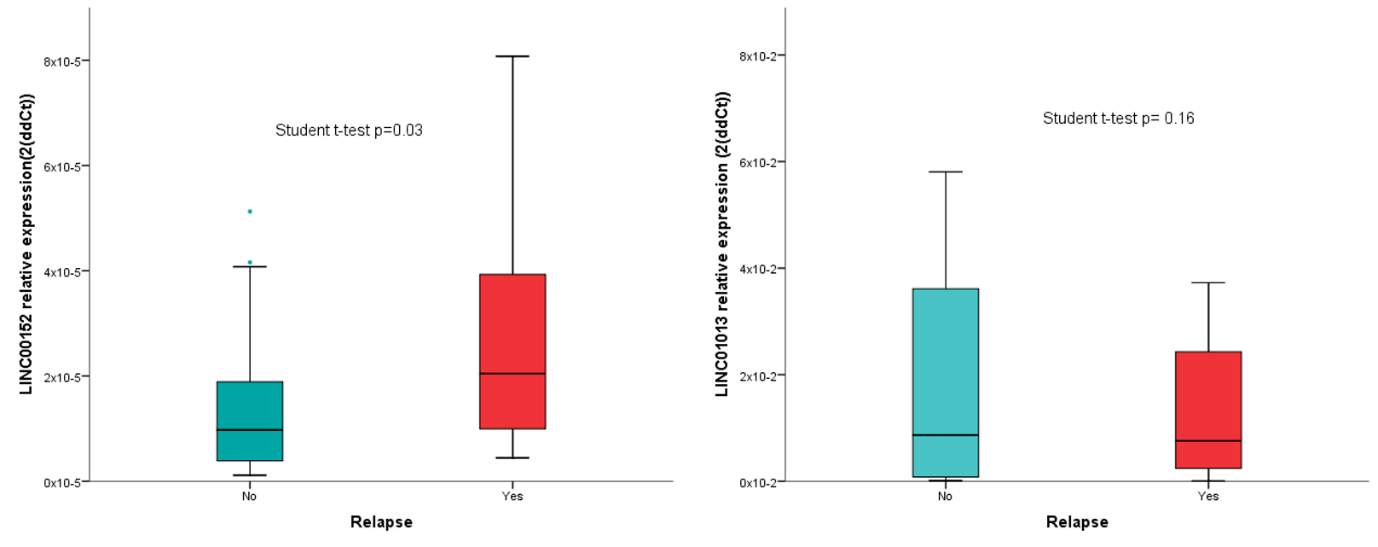


Supplementary Figure 2. Quantitative PCR (qPCR) analysis. Relative expression levels of *LINC00152* and *LINC01013* normalized to *SCARNA5*. The expression level of LINC00152 was significantly up-regulated in the relapsed group (p < 0.01). Although *LINC01013* was down-regulated in patients with relapse, no statistical significance was observed.
